# Complete genome sequence of *Schaalia odontolytica* isolated from subgingival biofilm

**DOI:** 10.1186/s12863-023-01184-9

**Published:** 2024-02-09

**Authors:** Eun-Young Jang, Jeewan Chun, Kyu Hwan Kwack, Ji-Hoi Moon, Jae-Hyung Lee

**Affiliations:** 1 Department of Oral Microbiology, College of Dentistry, Kyung Hee University, Seoul, 02447 Republic of Korea; 2 Department of Dentistry, Graduate School, Kyung Hee University, Seoul, 02447 Republic of Korea

**Keywords:** *Schaalia odontolytica*, Genome, Subgingival biofilm

## Abstract

**Objective:**

Recent advancements in genome-based taxonomic classification propose the reclassification of certain *Actinomyces* species into new genera, including *Schaalia*. *Schaalia odontolytica*, the type species within this genus, is frequently found in the human oral cavity and has been associated with actinomycotic lesions. Currently, only two complete genomes of *S. odontolytica* strains have been reported. Recognizing the limited research on subspecies-level variation of *S. odontolytica*, we conducted genome sequencing of strain KHUD_008, isolated from a Korean periodontitis patient’s subgingival biofilm. Additionally, we performed a comparative genome analysis using previously sequenced genomes of strain XH001 and strain FDAARGOS_732, both derived from the human oral cavity.

**Data description:**

Pacific Biosciences Sequel II sequencing generated 15,904 and 76,557 raw sequencing sub-reads, which were integrated to assemble the *de novo* genome using the Microbial Genome Analysis pipeline in the Single-Molecule Real-Time Analysis. The genome assembly completeness, assessed by Benchmarking Universal Single-Copy Orthologs, reached 99.2%. The genome is 2,389,595 bp with a GC content of 66.37%, and contains 2,002 protein-coding genes, 9 rRNAs, and 48 tRNA. Comparative analysis with two previously sequenced strains revealed many strain-specific genes in KHUD_008, primarily related to envelope biogenesis and replication/recombination/repair processes.

## Objective

In the human microbiome, the phylum *Actinobacteria* encompasses more than 30 genera, some of which have the potential to cause human actinomycosis, a chronic granulomatous infectious disease [1–3]. *Actinomyces israelii* (originally named *Streptothrix israeli*) was identified as the etiological agent responsible for actinomycosis by Kruse in 1896 [2, 4]. Other *Actinomyces* species, including *A. naeslundii*, *A. odontolyticus*, and *A. viscosus*, have also been implicated in actinomycotic lesions in humans [2]. However, recent advancements in genome-based taxonomic classification of the phylum *Actinobacteria* have indicated the need for revising several orders, families, and genera, as well as numerous species and a few subspecies [5]. As part of this reclassification, certain *Actinomyces* species are proposed to be moved into new genera, such as *Schaalia*. For example, two *Actinomyces odontolyticus* species strains, XH001 [6] and F0309 [7], most studied as references, were moved into *Schaalia odontolytica*.

The genus *Schaalia* comprises aerobic Gram-positive organisms that typically exhibit straight to slightly curved rod-shaped cells, although some species may produce coccoid or coccobacillary cells [5]. Their peptidoglycan composition includes L-lysine and N-acetylated muramic acid [5]. The genomic G + C content of *Schaalia* species ranges from 56 to 70%. The type species within this genus is *Schaalia odontolytica*, which commonly inhabits the oral cavity [3, 5]. Currently, only two complete genomes of *S. odontolytica* strains have been reported. Recognizing the limited research on subspecies-level variation of *S. odontolytica*, we conducted genome sequencing of strain KHUD_008, which was isolated from the subgingival biofilm of a Korean patient with periodontitis.

## Data description

The bacterial strain KHUD_008 was obtained by culturing subgingival bacterial biofilms of a Korean patient in Trypticase soy agar with defibrinated sheep blood under anaerobic conditions at 37 °C. Genomic DNA was extracted using Wizard HMW DNA Extraction Kit (promega). The complete 16 S rRNA gene sequence (~ 1.5 kb) was amplified using universal eubacteria primers (forward primer: 5’-AGAGTTTGATCCTGGCTCAG-3’; reverse primer: 5’-GGTTACCTTGTTACGACTT-3’) [8]. After purifying and sequencing the PCR product, species identification was performed *via* a BLAST search against the GenBank database.

For whole genome sequencing of *S. odontolytica* KHUD_008, a Single-Molecule Real-Time (SMRT) bell library was prepared following the manufacturer’s instructions (Pacific Biosciences). Two sequencing runs were performed, yielding 15,904 and 76,557 raw sequencing subreads with average read lengths of 8,559 and 8,610 bp using Pacific Biosciences Sequel II with 2.0 sequencing chemistry (Data file 1). The *de novo* genome of strain KHUD_008 was assembled using the Microbial Genome Analysis pipeline in the SMRT Analysis version 11.0 (https://www.pacb.com/support/software-downloads/) with default parameters, incorporating two sequencing run datasets. The NCBI Prokaryotic Genome Annotation Pipeline was employed for gene annotation.

The genome of *S. odontolytica* KHUD_008 is 2,389,595 bp long with a G + C content of 66.37% (Data file 2). It contains 2,002 protein-coding genes, 9 rRNAs (5 S, 16 S, 23 S), and 48 tRNAs. To assess genome completeness, Benchmarking Universal Single-Copy Orthologs (BUSCO) v5.4.7 [9] was used with “bacteria_odb10 dataset” (number of genomes: 4085, number of BUSCOs: 124), resulting in a genome assembly completeness of 99.2% (Data file 3). Additionally, we conducted a comparative genome analysis with previously sequenced genomes of strain XH001 and strain FDAARGOS_732, both isolated from the human oral cavity. This analysis revealed 103 strain-specific genes in KHUD_008, excluding unclassified and “unknown” genes. Strain-specific genes were found to be enriched in COG L (replication, recombination and repair) and COG M (cell wall, membrane, envelope biogenesis) categories (Data file 4).

We believe that the dataset, presented alongside the complete genome of *S. odontolytica* KHUD_008, forms a solid foundation for future studies on the genomic, transcriptomic and phenotypic characterization of this species, as well as other closely related species. As the third publicly available complete genome sequence of *S. odontolytica*, this data will be a valuable resource for comparative genomics, enabling more comprehensive analyses and discoveries regarding strain-level variation. Table 1 provides the links to Data files 1–4 and Data sets 1–3.

**Table 1:** Overview of data files/data sets

Label	Name of data file/data set	File types (file extension)	Data repository and identifier (DOI or accession number)
Data file 1	Subread length distribution of sequencing reads	Portable Data Format file (.pdf)	Figshare (10.6084/m9.figshare.23591493) [10]
Data file 2	Genome features of *Schaalia odontolytica* KHUD_008	Portable Data Format file (.pdf)	Figshare (10.6084/m9.figshare.23591523) [11]
Data file 3	Short BUSCO summary	Portable Data Format file (.pdf)	Figshare (10.6084/m9.figshare.23591538) [12]
Data file 4	Strain KHUD_008 specific genes belonging to COG L and COG M	Portable Data Format file (.pdf)	Figshare (10.6084/m9.figshare.23591544) [13]
Data set 1	Sequencing read dataset 1 of *Schaalia odontolytica* KHUD_008	Fastq file (.fastq.gz)	NCBI Sequence Read Archive (https://identifiers.org/ncbi/insdc.sra:SRX19887473) [14]
Data set 2	Sequencing read dataset 2 of *Schaalia odontolytica* KHUD_008	Fastq file (.fastq.gz)	NCBI Sequence Read Archive (https://identifiers.org/ncbi/insdc.sra:SRX19887474) [15]
Data set 3	Genome assembly of *Schaalia odontolytica* KHUD_008	FASTA / GenBank / ASN.1	NCBI Genome assembly (https://identifiers.org/ncbi/insdc.gca:GCA_024584435.1) [16]

## Limitation

Initially, we obtained the sequencing subread data using Pacific Biosciences Sequel II, and the number of the subread was less than 20,000. In order to obtain a more comprehensive dataset, we performed an additional sequencing run using the same sequencing library, resulting in over 70,000 subreads. By combining the datasets from both sequencing runs, we successfully generated the complete and circular genome for *S. odontolytica* KHUD_008. Therefore, we believe that there are no limitations in terms of data acquisition and the complete genome assembly process in this study.

## Data Availability

Data files 1–4 described in this Data note can be freely and openly accessible on Figshare (https://figshare.com/) [10–13]. Data sets 1–3 are available on the NCBI database. The raw reads have been submitted to the NCBI Sequence Read Archive under the accession number SRX19887473 [14] and SRX19887474 [15] (Data set 1 and 2). The genome assembly of the *S. odontolytica* KHUD_008 was submitted to NCBI GenBank and are available under the accession number GCA_024584435.1 (Data set 3) [16].

## Abbreviations

BLAST: Basic Local Alignment Search Tool
SMRT: Single-Molecule Real-Time
NCBI: National Center for Biotechnology Information
BUSCO: Benchmarking Universal Single-Copy Orthologs
COG: Clusters of Orthologous Groups of proteins
